# A systematic review of risky-choice framing effects

**DOI:** 10.17179/excli2023-6169

**Published:** 2023-09-19

**Authors:** Anton Kühberger

**Affiliations:** 1 Department of Psychology & Centre of Cognitive Neurosciences, University of Salzburg, Austria

**Keywords:** choice, framing, prospect theory, risk

## Abstract

Classic decision theory requires that rational agents show description invariance: which description is chosen should not matter for judgments, preferences, or choices given the descriptions are co-extensive. Framing research has amply demonstrated a failure of description invariance by showing that the choice of the description has a systematic effect on judgments, preferences, and choices. Specifically, framing research has shown that linguistically different descriptions of seemingly equivalent options frequently lead to preference reversals. I summarize the research on framing in situations entailing risk. This includes the characterization of different research designs used, the size and robustness of the framing effects reported for those designs, and the theoretical accounts put forward to explain framing effects. The theoretical accounts are evaluated with respect to their merits, empirically and theoretically. I end by providing the implications of framing research. My central point is that the existence of framing effects points to the adaptiveness of the processes underlying human judgment and choice rather than simply showing human irrationality.

## Introduction

Language is imprecise. Therefore, there are different ways of saying the same thing. For instance, rather than saying that “Tom told me the truth about X”, I could also say “Tom did not lie about X”. These are just two ways of saying the same thing, and there may be others. But is telling the truth really the same as not telling a lie? If your glass of beer is half full, it is, by implication, also half empty. Would you be indifferent between these two descriptions, in all cases? If you were thirsty, wouldn't you prefer to say that the glass is half empty? In contrast, if you were satiated, wouldn't you describe it as half full? Ground beef containing 80 % lean also contains 20 % fat: would you pay the same price for ground beef described as containing 80 % lean as compared to 20 % fat? 

Research on the effects of communicating similar things differently as in the examples above is referred to as framing research. When framing situations, people use different words for communicating some identical objective reality. That is, the difference lies in the words used rather than in the underlying reality. Framing research shows that seemingly innocent differences in wording can have important consequences. Here I focus on the method of framing traditionally referred to as *risky choice framing*. I will briefly introduce the basic concept of risky choice framing and will give an overview about how it is manipulated in different fields. Then I will discuss the literature, beginning with describing the landmark study in the field. I follow by an overview of accounts attempting to explain why framing effects occur. I conclude with a discussion of the broader implications of understanding risky choice framing for decision-making in general.

## Risky Choice Framing: The Classic Problem

The insight that some state of affairs can be described differently - i.e., that it can be framed - is in itself not very surprising. What makes the issue interesting is that, in many cases, the difference in description - the framing - matters for judgment and choice, and that there is some regularity in its effect. In short, framing research shows that the choice of the description of a situation has predictable effects on cognition. The framing effect is a reliable empirical finding, reported in hundreds of papers since the first investigation into risky choice framing in the early 1980ies by Tversky and Kahneman (1981[75]). They presented half of their experimental participants with the following task, which later became known as the “Asian Disease Problem” (ADP). The ADP is as follows:


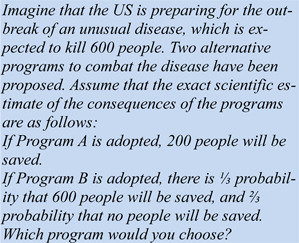


Confronted with this task, participants showed a marked preference: about 75 % preferred Program A, saving 200 people. This is somewhat surprising from the standpoint of traditional decision theory. Note that a backbone of traditional decision theory is that preferences should be driven by utilities. In situations that can be modelled after gambles, the rational choice is prescribed by its subjective expected utility (SEU). The SEU of a gamble is roughly calculated by summing up the products of probability and payoff of the individual options: *SEU* = Σ(*p* • $). In the case of the ADP above we get for Program A: SEU(A) = 1 x 200 = 200. For Program B we get SEU(B) = [(⅓ x 600) + (⅔ x 0)] = 200. That is, we end up with identical utility, and participants therefore should be indifferent between the programs (Note that indifference was not allowed in Tversky and Kahneman's experiment and is also not allowed in most studies following their lead). The SEU model is, however, not challenged by this finding, since utilities need not be identical to the numerical values and one can easily derive an utility function that allows the preference for Program A over Program B.

The ingenious manipulation by Tversky and Kahneman (1981[75]) in the ADP is, however, that exactly the same situation can be framed differently. Here it is: 


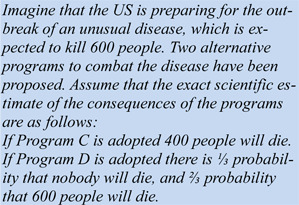


Confronted with this task, participants also showed a marked preference: about 75 % preferred Program D, (⅓ probability that nobody will die, and ⅔ probability that 600 people will die) over Program C (400 people will die). Again, the deviation from indifference is surprising, but can be described by assuming some specific utility function. Note, however, that options A and C are describing the exactly same outcome (saving 200 out of 600 means that 400 people out of 600 are not saved), as do options B and D (saving 600 with ⅓ probability is identical to loosing 600 with ⅔ probability). With such a pattern of preferences SEU runs into problems because utility is defined over objective states of wealth, and thus the objective outcome matters. Note that the outcome is identical in all 4 programs: 200 people are surviving the disease. If people's choices were driven by utility, they should be consistent: those preferring Program A over B should also prefer Program C over D. And those preferring B over A should consequently prefer D over C. The actual pattern of findings was different, however: a majority preferred A over B, but also preferred D over C. Explaining this reversal of preferences, in this gamble-like problem, which came to be known as the *risky-choice framing effect*, became the topic of a huge research endeavour in the following years.

### Description invariance

The basic construal was that, in the ADP, participants are confronted with two objectively identical options that are described as if they were different, namely as gains (Programs A and B) or as losses (Programs C and D). The difference between the programs within each frame was conceptualized as a difference in risk, with A and C being sure (or riskless) options, and B and D being risky options. The typical finding, that with positive framing (gains) participants tend to prefer the sure option, while they prefer the risky option with negative framing (losses), is the risky choice framing effect. This effect appears to be entirely irrational on logical grounds. Indeed, framing research gained its momentum from the interpretation that it violates a basic normative principle, frequently called *description invariance* (or extensionality) principle: “Different representations of the same choice problem should yield the same preference. That is, the preference between options should be independent of their description” (Tversky and Kahneman, 1986[74], p. 253). Description invariance thus requires that variations of form should not affect judgment and choice, as long as the actual outcomes are identical. This appears to be rather obvious, but it fails in many cases. Examples other than the risky choice task are: If item X costs $10 more than item Y, then, by implication, Y is $10 cheaper than X: Would this difference in description influence your choice? Who fared better (or worse): the person who succeeded in solving half of the questions of a test vs. the person who failed in solving half of the questions? If your glass is half-full, is it half-empty at the same time? A sample consisting of a minority of females, contains, at the same time, a majority of males. Some cheap item might be tagged $ 1.99 or ¢199. Does this matter for buying? Is the incidence rate of 0.1 in 100 less threatening than 1 in 1,000? 

After calling attention to framing, we see framing processes at work nearly everywhere. The ubiquity of framing processes offers good reason for researchers from different disciplines investigating the phenomenon. Here we focus specifically on risky choice framing a la ADP. The framing effect - risk aversion with gains and risk-seeking with losses - has proven robust in hundreds of empirical examinations. This effect became the posterchild of human irrationality not only in psychology, but also in economy, philosophy, and linguistics. I will argue that this irrationality message is inappropriate.

## Framing Tasks

The ADP has been used in many variations, from nearly identical to considerably different. Kühberger (1998[36]; Steiger and Kühberger, 2018[71]) collected many of the variations in operationalization, design, and procedure. Those include features of risk (how is the risk manipulated; how much the options differ in risk; how many risky events are included); design (the nature of the framing manipulation, response mode, between vs within subjects manipulation); and task domains (gambling, health, business, social). Meta-analysis showed that most variations influenced the size of the framing effect, but the differences were not dramatic (Steiger and Kühberger, 2018[71]). That is, the effect-size varied, but the effect did not disappear.

### Varieties of framing tasks

Note, however, that some framing tasks differ from the classic ADP in an important respect: their options are not equivalent. For such tasks the principle of description invariance does not apply, and framing effects seen in such tasks have not been considered irrational. This basic distinction, concerning the equivalency of options, was proposed by Druckman (2004[16]). In the ADP the options are considered equivalent (I will return to the issues revolving around the equivalence of frames later). Much framing research uses tasks that quite obviously do not contain equivalent options. Rather, this research uses frames that differ in emphasis. *Emphasis framing* highlights a subset of potentially relevant considerations. The example Druckman (2004[16]) gives is the act of giving assistance to the poor by the government. This can be framed either as a humanitarian act, or as a government expenditures problem. A single issue is framed here by highlighting its different, no co-extensive, aspects. Recently Bermúdez (2022[4]) has argued along similar lines, using an example from a Greek play. In the plot Agamemnon faces the prospect of sacrificing his daughter, Iphigenia, to the goddess Artemis, for saving the inhabitants of Troy. If Agamemnon is killing Iphigenia, this could be framed in either of two ways: as (i) following Artemis's will, or (ii) murdering his daughter. The act of killing is identical under both frames, but the different frames imply distinct reasons for acting one way or another. According to Bermúdez (2022[4]) it can be perfectly rational to evaluate something differently under different descriptions, even while recognizing that the descriptions are equivalent. One need not follow this argument, however, especially in equivalency framing tasks. For instance, if somebody describes a project's chances to be 70 % success, rather than 30 % failure, it presumably is more difficult to rationally defend different preferences. Evaluating the amount of research devoted to emphasis framing as compared to equivalency framing, Borah (2011[8]) counted the number of published framing studies between 1997 and 2007 and found about 60 % of 380 papers using emphasis framing, and only about 20 % using equivalency framing. Emphasis framing is a topic heavily investigated in communication research and it involves presenting general or specific issues - often political ones - in a specific context, thus emphasizing different aspects. Emphasis frames thus fail to be logically equivalent. 

### Varieties of equivalency framing tasks

The ADP is an instance of *equivalency framing*. A well-known typology of equivalency framing tasks was proposed by Levin et al. (1998[44]). They distinguished among *attribute framing*, *goal framing*, and *risky-choice framing*. In attribute framing a single attribute is framed (e.g., x % success vs. (1-x) % failure; y % lean vs. (1-y) % fat; z % of patients experience side effects vs. (1-z) % do not experience side effects). Most researchers (e.g., Levin and Gaeth, 1988[43]) are favoring as an explanation for attribute framing an associative priming account whereby the positive or negative labels evoke similarly valued associations. Investigation of the literature results in the impression that the effect of attribute framing is robust and reliable. Recent research seems to indicate that the effect is mainly driven by cognition, as opposed to affect. Thus, if people are allowed to gain consumption experience in addition to, or instead of, purely hypothetical framed information, the attribute framing effect diminishes or disappears (Poor and Isaac, 2023[57]). I am not aware of a meta-analysis providing an overall picture of the size and heterogeneity of attribute framing effects, however.

Goal framing consists of a more complicated procedure. It attempts to highlight either the positive or negative consequences of performing, or of failing to perform, an act. Thus, the framing consists in focusing attention either on attaining the positive, or preventing the negative, outcome, by focusing on (i) doing or avoiding something that is (ii) desirable or undesirable. Classic explanations of goal-framing effects are inspired by *Prospect theory* (PT, see below) and its idea of *loss aversion* (Rothman and Salovey, 1997[63]), which were later (Rothman et al., 2008[64]) enriched by appeals to self-regulation. The proposal is that self-regulation can be guided by prevention goals (being concerned with safety and security; the fulfilment of obligations; the absence of unfavorable outcomes), or promotion goals (being concerned with accomplishments and ideals; the presence of favorable outcomes; Higgins, 2000[24]). A promotion-focused mindset, so it is argued, renders the presence or absence of favorable outcomes especially salient and persuasive. In contrast, a prevention-focused mindset renders the presence or absence of unfavorable outcomes especially salient and persuasive. Although this explanation appears plausible, there is considerable doubt on the existence of goal framing effects. Meta-analyses (Gallagher and Updegraff, 2012[18]; Nabi et al., 2020[51]; O'Keefe and Jensen, 2008[52]; 2009[53]) report very small, and mostly insignificant, effect sizes. 

### Prospect theory

The leading explanation of risky choice framing effects is based on Prospect Theory (PT; Kahneman and Tversky, 1979[29]). The theory proposes two deviations from classic EU-theorizing: that outcomes are coded relative to a reference point which, unlike in classic theorizing, is unlikely to be zero, and that probabilities are translated into decision weights, rather than being processed linearly. According to PT valuation depends on the reference point: In the domain of gains, the value function *v* is concave, while, in the domain of losses, it is convex. In addition, PT holds that the slope of the value function is steeper for losses than for gains. Therefore, the effect of losses is stronger than the effect of gains, leading to loss aversion. Loss aversion is the tendency to feel greater discomfort and making riskier decisions when faced with losses than with gains. In addition, PT proposes that outcomes near the reference point are getting disproportionally high value. This follows from the well-known notion of diminishing utility, which is dating back to 1738 in the work of Daniel Bernoulli (1738/1954[5]) and his article "Exposition of a New Theory on the Measurement of Risk." 

Applying the ideas of PT to the ADP leads to the following reasoning: in the gain domain (i.e., when the options appear to be gains; in reality they can be losses; note that in the ADP there is nothing to be gained but lives can only be lost) the concavity of the value function renders a small sure gain more attractive than a larger risky gain since v(+200) > [⅓v(+600) + ⅔*v*(0)]. Note that, since the value of 0 is assumed to be 0 (see Reyna and Brainerd, 2023[59]; Schulte-Mecklenbeck et al., 2017[66], for evidence that this assumption is unrealistic), the choice is between *v*(+200) (the sure gain), and ⅓*v*(+600) (the risky gain). Due to the concavity for gains of the value function* v*(+200) > ⅓*v*(+600), and people prefer the sure gain. In the domain of losses the matter is different: the value function for losses is convex, rendering a large risky loss less unattractive than a small sure loss: v(-400) < ⅔v(-600). The choice then is between *v*(-400) and [⅔*v*(-600) +⅓*v*(0)]. Since the value function for losses is convex, the risky loss is less aversive and is therefore preferred over the sure loss. We end up with the framing effect: risk aversion with gains, and risk seeking with losses. This pattern is relatively stable over different types of valuable outcomes like lives, or money.

**Figure 1:**
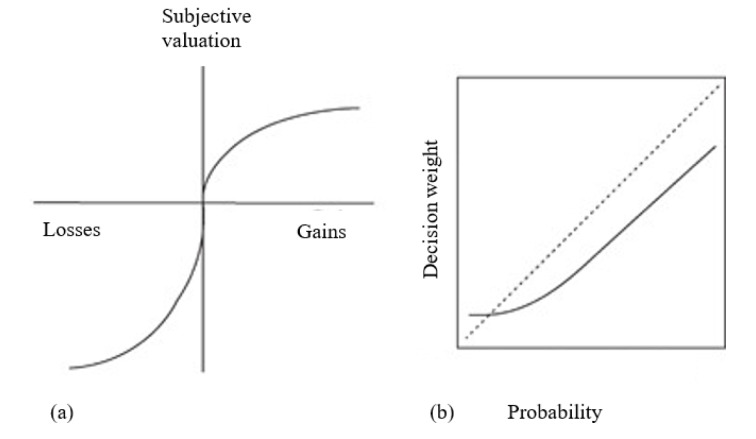
Schematic functional forms of Prospect Theory. (a) Value function. (b) Probability weighting function

The explanation based on PT is the dominant cognitive explanation of risky-choice framing effects. A variety of related explanations take the weighting of probabilities also into account. For instance, Cumulative prospect theory (Tversky and Kahneman, 1992[73]) predicts a different pattern: risk aversion for gains and risk seeking for losses of high probability, but risk seeking for gains and risk aversion for losses of low probability. Here risk attitude is determined jointly by outcome valuation and probability weighting. Venture theory (Hogarth and Einhorn, 1990[26]) also explains framing effects, and does this only by reference to probability weighting, but there is only little research based on the latter. Figure 1(Fig. 1) depicts the basic functional forms of PT: the value function for gains and for losses, and the probability weighting function over the range of probabilities.

## The Size of Framing Effects

### Model testing in framing research

I have argued for a risky choice framing effect. What does this mean? In general, an effect can be said to exist if robust positive evidence is provided in empirical studies. That is, if studies succeed in showing a significant effect, thus rejecting the null hypothesis of no effect. If most (published) studies report a significant effect, and more importantly, if the effect is reliably found in meta-analyses, the scientific community tends to take the effect for granted. What, however, should be concluded if approximately a quarter of the participants in framing studies do not show a framing effect? Recall that in the classic experiment by Tversky and Kahneman (1981[75]) about 75 % of the participants showed risk-aversion with gains, and about 75 % also showed risk-seeking with losses. These participants showed the effect, the remaining 25 % did not show a framing effect. Curiously, however, hardly anybody seems to care about this prediction error. PT is appropriate because, so the argument goes, it predicts better than SEU, which predicts no framing effect (i.e., 50:50 choices). Although this interpretation may be warranted from the perspective of standard significance testing, it is not uncontested. Standard significance testing takes the 50:50 split as the null-hypothesis. Note that, in the case of equivalent options, this is not a nil prediction, but rather a prediction that follows from a specific theory. By doing a significance test against the 50:50 prediction SEU is taken for granted, and a significant finding is interpreted as being inconsistent with SEU. Another hypothesis must then be true, and by this reasoning PT wins. This test is unfair, by being biased against SEU. Imagine that we flip the reasoning around by taking PT for granted. That is, we are testing whether our findings significantly deviate from the predictions of PT. Lacking an error theory, PT predicts that all participants will prefer the sure option with gains, and all will prefer the risky option with losses. Recall that the finding was that about 75 % preferred the sure gain, and 75 % preferred the risky loss. Doing a significance test against the 100:0 prediction would again result in a significant effect, since both percentages do also deviate from 100 %. We thus would reject PT, and by lack of another candidate, would accept SEU, or any other unspecified model. 

We see that simple significance testing does not do the job. What we need to know is whether PT predicts better than SEU in a direct test, a question that cannot be answered by traditional significance testing. To provide such a test, the predictions of the candidate theories ought to be much more specific. In other words, PT lacks an error model, which would provide an estimate of the amount of variation to be expected. This also applies to SEU, however, since equivalency in EV does not entail equivalency in SEU. Indeed, utility theory allows for various reasons for preferring one option over another, in addition to EV. To my knowledge, error models are not existing, for either theory. By the way, this is a critique that applies to most psychological theories: that they are quite unspecific as to what exactly they are predicting. Rejection of some null-hypothesis in a significance test does not automatically mean that the alternative hypothesis is true. Significance thus is not an unequivocal yardstick for accepting hypotheses. A recent study takes this criticism serious: Huizenga et al. (2023[27]) correctly point out that an adequate theory of framing not only needs to describe the framing effect proper, but also should account for context-related, task-related, and individual differences that are a source of variance. In a most laudable effort they formalize the predictions of four models of the risky choice framing effect and test their predictions against each other. The result is that the majority of decision makers decide according to some hybrid model, incorporating ideas from different models. Thus, THE THEORY on risky choice framing probably does not exist.

### Effect sizes

Putting aside the issue of significance testing, we can ask what the size of the effect is in terms of percentages preferring the sure over the risky gain, and the risky over the sure loss. This question is best answered in meta-analyses. There are meta-analyses of framing effects in general, failing to provide effect sizes corrected for potential publications bias (e.g., Kühberger, 1998[36]) or providing estimates that are corrected for publication bias (e.g., Steiger and Kühberger, 2018[71]). In addition, there are meta-analyses that distinguish among attribute, goal, and risky choice framing (e.g., Levin et al., 1998[44]), meta-analyses specifically on message-framing (e.g., Gallagher and Updegraff, 2012[18]; Nabi et al., 2020[51]), and meta-analyses on risky-choice framing effects (Gong et al., 2013[20]; Kühberger et al., 1999[39]).

As discussed above, it is theoretically unclear whether we should expect a 50:50 choice split in risky choice framing tasks. Empirically, this question can be answered by providing an “unframed” condition, i.e., a condition framed in both positive and negative terms. Druckman (2001[15]) did such a study. His sure option was described as “200 people will be saved and 400 people will die.” That is, the positive as well as the negative aspect of the sure option was described. In this unframed task the framing effect disappeared (see also Huizenga et al., 2023[27]). Although it is unclear whether this was due to the provision of both frames (saved, die) or due to the provision of both outcomes (200, 400), we might take this as preliminary evidence that the even split is an appropriate yardstick for measuring framing effects.

Alternatively, since risky choice framing experiments present two different framing conditions, we could also test for a difference between conditions, rather than for a deviation from 50:50. Note that these tests can result in different conclusions. Kühberger (2022[32], p. 66 ff) gives the following example to exemplify this case: Imagine a risky-choice framing experiment with 30 participants in either framing condition. The result of the experiment is that 10 participants (33 %) prefer the sure option in gain framing, whereas with losses, only 3 (10 %) prefer the sure loss. The statistical test for a difference between conditions yields χ^2^(1, N=60) = 4.81, *p *= .029, showing significantly more risk seeking with losses than with gains. This unidirectional framing effect, defined relative to two different framing conditions, indicates increased risk seeking in the negative framing condition in relation to the positive condition. This is what PT predicts, isn't it? Nevertheless, one could disagree because PT specifically predicts risk aversion for gains, and risk-seeking for losses. Evaluated from the perspective of the even split, this means that more than 50 % of the choices are for the sure gain, and that more than 50 % of choices are for the risky loss. In the above example such a test would result in a failure to find a framing effect for gains (10 of 30 fails to be higher than 50 %, [χ^2^(1, N=30) = 3.33, *p* = .068]; there is even a tendency in the wrong direction), but a strong effect for losses (χ^2^(1, N=30) = 19.2, *p* < .0001). We end up in a predicament: the same finding can be said to show (i) a significant framing effect (the comparison of two conditions is often called the unidirectional test); (ii) no framing effect for gains but a framing effect for losses (in the bidirectional test). Does such a finding support PT, or does it contradict the theory?

Regardless of the answer to this question, there are meta-analyses evaluating the size and existence of framing effects. Probably the first comprehensive meta-analysis was done by Kühberger (1998[36]). He summarized over 100 studies containing well over 200 single effect sizes collected on samples of about 30,000 participants overall. He reported a weighted (by sample size) mean unidirectional (i.e., between framing conditions) effect size of Cohen's *d* = .31. Meta-analyses suffer from possible publication bias, however. Publication bias is a variety of the more general reporting bias, pertaining to the selection of manuscripts for publication in peer-reviewed scientific outlets. Publication bias means failure of publishing a scientific finding because the result falls short of the traditional significance level of p < .05 (see Kühberger, 2023[33], for a bibliographic review). If this applies for a phenomenon, the published research is systematically unrepresentative of the existing research, and, due to the selectivity based on significance, overestimates the true effect size. Therefore, it became accepted practice to correct for possible publication bias in meta-analyses. A variety of methods for correction are existing, and there is ongoing discussion on their strength and weaknesses (see Kühberger, 2023[33]). Kühberger's (1998[36]) meta-analysis was therefore repeated and extended 20 years later by Steiger and Kühberger (2018[71]), correcting for possible publication bias. This new analysis reported - somehow surprising - a corrected effect size of *d* = 0.52. Upward correction implies no publication bias in framing research.

The corrected estimate appears to be quite robust, considering different other sources. First, it is very similar to the small meta-analysis that was done by Steiger and Kühberger (2018[71]) on framing studies that were published in the year 2016. This analysis reported an effect size of *d* = 0.56. Second, the effect size reported in the *Many Labs Replication Project *(Klein et al., 2014[30]) on risky choice framing is *d* = 0.60. This effect size does not suffer from publication bias, since the results from all studies included in the Many Labs Replication Project were added up, not allowing for result-dependent selection. Third, a recent study using a large dataset from the COVIDiSTRESS Global Survey Consortium included a classic ADP framing task. The authors (Im and Chen, 2022[28]) reported an overall effect size of Cohen's *h* = 0.612, 95 % CI [0.568, 0.656] for samples from 49 different countries including over 100,000 participants. Cohen's h is calculated as the arcsine-transformed difference in two proportions, and is similar in size to Cohen's d, which is the standardized difference in two independent sample means, standardized by average variance. In sum, the risky choice framing effect as measured by the ADP is about d = 0.60. To provide a grip on the magnitude of such an effect, the Binomial Effect Size Display (Rosenthal, 2005[62]) is helpful. It displays the difference between two proportions in an intuitive manner, based on the correlation coefficient as the effect size measure. Cohen's d is easily approximated as a correlation coefficient by


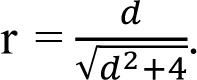


Thus, d = 0.60 translates into r = 0,28. The Binomial Effect Size Display shows that such an effect is equivalent to increasing the success rate from 36 % to 64 %.

Note, however, that there is no single “true effect size” for risky choice framing tasks, since tasks and contexts differ, even when modeled after the classic ADP. The numbers involved may differ (although 600 seems to be a magical number, since by far most studies use it), the probabilities involved may differ (again, ⅓ and ⅔ are mostly used), the topics may differ (health, money, time, etc.), and the studies may differ with respect to a variety of methodological and design features (e.g., single vs multiple choices, between vs within manipulation, or how the risk is manipulated). Steiger and Kühberger (2018[71]) reported that these variations led to differences in effect size ranging from a minimum size of d = 0.16 to a maximum of d = 0.91 (see their Tables 1 and 2).

For the subgroup of ADP-like risky-choice framing tasks, a meta-analysis (Kühberger et al., 1999[39]) on more than 40 studies reported 63 % choices of the sure gain, and 41 % choices of the sure loss. That is, the difference was 22 %.

A recent study is especially interesting in answering the question of how much heterogeneity can be found in the ADP. The authors (DeKay et al., 2022[12]) conducted a metastudy on framing. A metastudy contains a set of microstudies that are created from a subset of all possibilities, thus combining some of the advantages of meta-analysis and replication. Specifically, in their framing Study 1 they included variations in domain (human lives; money; animal lives; crops), magnitude of outcome (600 human lives; $15,000 of investment; 3,600 animals; 24,000 acres of crops: each multiplied by 0.1, 0.5, 2, or 10), probability (13 different values), probability format (expressed as ⅓ probability; rounded percentage -33 % chance -, or words - a one-third probability), expected value (also two unequal options were used), risk comparison (the certain option replaced by a slightly risky option), option order (sure-risky vs risky-sure), and layout (expanded vs. compressed). They found that the framing effect generalized well over almost all those variables (called facets in their study), reaching about 26 percentage points on average. In sum, there is a striking similarity in estimated effect sizes over different methodological procedures and different statistical analyses. 

### Individual differences

It is obvious that numerous factors can have an effect on the framing effect. Beratsova et al. (2016[3]) enumerate four broader groups: task setup, experience, effort, and demographics. To begin with demographics, or individual differences, there indeed is some research investigating the role of individual differences for risky choice framing effects. Among the best researched individual difference dimensions are those that directly or indirectly relate to the interplay of two different systems of reasoning. One system, generically called “System 1,” is characterized by fast, intuitive, effortless processing, often identified by the reliance on heuristics. The second, called “System2,” is characterized by slow, deliberate, logical processing (e.g., Stanovich and West, 2000[70]). Researchers tended to assume that bigger framing effects occur when people rely on System 1 processing, due to external factors like time pressure (e.g., Diederich et al., 2018[13]; Roberts et al., 2022[60]). In contrast, when people are forced to deliberate in framing studies, the effect was shown to be smaller (e.g., Almashat et al., 2008[2]). Consequently, it was assumed that, if thinking styles related to System 1 vs System 2 are dispositional, such dispositions should also mediate the framing effect. A variety of such styles have been investigated, measured by a variety of inventories like the Rational-Experiential Inventory, the Need for Cognition scale, the Actively Open-Minded Thinking Scale, or the Faith in Intuition scale. Although significant findings are reported here and there, no consistent picture emerges from this research. The findings are probably best summarized by Wyszynski and Diederich (2023[78]): that there is no consistent relationship between the susceptibility to framing and cognitive styles or risk styles. Even numeracy shows only little effects in framing studies (Peters and Levin, 2008[55]). Matters are similar with respect to effort, experience, and nationality.

When it comes to risk-taking, age is always an interesting variable. Indeed, there are studies investigating the influence of age on framing. In a meta-analysis Best and Charness (2015[6]) investigated the pool of studies for both positively and negatively framed conditions, including data from 18 studies and more than 3,000 participants. They found a tendency for younger adults to be more risk-prone than older adults in positive framing, specifically with small-amount financial and large-amount mortality-based scenarios. No effect of age was found for negatively framed problems. Thus, if age differences exist, they are small at best. A similar conclusion presumably pertains to effects of the ”big five” personality traits: here I take the absence of conclusive evidence as conclusive evidence for the absence of an influence.

With message framing tasks the evidence for a framing effect is weak, at best. Earlier work (O'Keefe and Jensen, 2008[52], 2009[53]) reported small effect sizes of framing on message engagement and persuasiveness of about *d* = 0.10. Recent meta-analyses on message framing in specific domains also report mixed findings. For instance, Florence et al. (2022[17]) investigated message framing effects on sustainable consumer behavior. In their analysis twelve studies reported positive framing being more effective, and 10 studies reported higher effectiveness for negative framing. A meta-analysis on the effects of message framing on cancer prevention and detection behaviors, intentions, and attitudes (Ainiwaer et al., 2021[1]) including 24 randomized controlled trials also reports sobering findings: loss-framed messages improved cancer detection behaviors somehow in the short run, but framing was ineffective for attitudes and intentions for cancer prevention and cancer detection. The gloomy conclusion of the authors is that it is almost impossible to influence people by gain or loss-framed messages. Overall, the evidence for message framing effects is hardly decisive and authors should avoid statements indicating that loss-framed statements are effective under conditions of risk.

For attitude framing nearly no evidence exists in terms of meta-analysis. Maybe this is due to the fact that describing something in positive terms (e.g., x % lean) should, on face validity, lead to better evaluations than doing it in negative terms (1-x % fat). I currently know of one meta-analysis investigating framing effects on consumers' attitudes and intentions toward food. The meta-analysis (Dolgopolova et al., 2021[14]) included 25 studies containing 76 effect sizes. The overall effect was that the gain frame resulted in people reporting higher attitudes and intentions than the loss frame, with an effect size of *d* = 0.47. However, a subgroup analysis showed, somehow surprising, that a strong framing effect existed for attitudes (d = 0.82), but not for intentions (d = 0.05). It seems that attitudes are more easily influenced, but with little downstream effects on intentions.

Summing up there is strong and consistent evidence for a framing effect in risky choice framing. Little, but mostly positive, evidence exists for an attribute framing effect. In contrast, there is a lack of consistent evidence for a goal or message framing effect. Note that this conclusion is based on a considerable amount of empirical work on the topic. 

## Explaining the Framing Effect

The empirical work on equivalency framing was originally inspired by PT, reported in a *Science* paper by Tversky and Kahneman (1981[75]). They had developed their PT in 1979 (Kahneman and Tversky, 1979[29]) as a refinement of SEU theory which better described human preferences. PT was influential in many domains within and beyond classic decision theory, and Daniel Kahneman got the Nobel Prize for Economy in 2002 not the least for this contribution. PT, as described earlier, was used to predict risky choice framing effects, especially to explain why people tend to prefer playing it sure with gains, while they are more risk-prone with equivalent losses. A central limitation for developing predictions from PT is, however, that risk being involved somehow. That is, the options need to differ with respect to risk, otherwise the effect of framing on risk attitude cannot be measured. However, not all framing experiments involve options that differ in risk. For instance, describing ground beef in terms of percent lean, or percent fat, results in a framing effect that cannot be predicted by PT. Such effects also need explanation, and PT does not yield here. Consequently, researchers came up with other explanations for framing effects, that, as it appears, do not only apply for riskless situations, but do also apply for classic risky choice framing tasks. PT thus got competitors. Currently an array of models is existing for framing effects: cognitive-formal, emotional-motivational, and communicative-pragmatical. 

### Cognitive-formal models

Cognitive-formal models are in some sense psychophysical, as they rely on different value- or weighting functions for gains, and losses. Often these functions are specified in a way allowing choices and preferences to be irrational. Thus, they can also serve as explanations for the existence of cognitive illusions.

Valuation and weighting are considered dependent on details of task and context. PT is of this kind. PT is cognitive, since it distinguishes three phases in decision making: first a translation phase, where outcomes are coded relative to a reference point, and probabilities translated into decision weights. Then, in the combination phase, values and weights are combined. Finally, in the decision phase, the prospect with the highest combination is chosen. As argued, PT's framing explanation is via subjective valuation by positing a reference point that separates gains from losses. The value function is different for gains and losses (in Tversky and Kahneman, 1992[73], the respective functions for gains and losses are *U*(x) = x^α^ for x ≥ 0, and *U*(x) = −λ(−x)^β^ for x < 0, with median parameters of α = β = 0.88, and λ = 2.25), and the slope of the value function is steeper for losses than for gains. This phenomenon, to feel the pain of loss more strongly than the pleasure of the equivalent gain is called loss aversion in decision theory. The strength of the aversion to loss compared to the attraction to gain is captured by the parameter, λ, of the value function for losses. Loss aversion has been widely applied in decision theory, economics, and beyond. A meta-analysis on over 600 empirical estimates of loss aversion between 1992 and 2017 finds a mean size of λ = 1.955. That is, losses have approximately twice the weight of gains (Brown et al., 2021[10]).

The idea of loss aversion as an explanation of framing phenomena, has been prominently formulated in Cumulative prospect theory (CPT; Tversky & Kahneman, 1992[73]). CPT refines PT by allowing for cumulative rather than separable probability weights. Importantly, this refinement led to a new prediction for framing: risk aversion for gains and risk seeking for losses - as usual - but only for high probability gambles. For low probabilities the reversal was predicted: risk seeking for gains and risk aversion for losses. That is, probabilities and their weights also come into play for predicting framing effects. In the meta-analysis of Kühberger et al. (1999[39]) this prediction was tested for risky choice framing tasks and was only partially supported. Specifically, there was no reversal of the classic framing effect with low probabilities. A similar finding is reported in Huizenga et al. (2023[27]), who do also offer a formalization of the separate steps articulated in CPT.

Some accounts related to PT and CPT have been applied to risky choice framing, accruing less empirical work, however. They differ mostly with respect to the formal form either of the value, or of the weighting function. For instance, *Venture theory* (Hogarth and Einhorn, 1990[26]) predicts framing effects based on the probability weighting function alone. A model called *The Advantage model *(Shafir et al., 1993[67]) also predicts framing effects but it allows for a more complicated pattern of the weighting of payoffs and probabilities. Overall, however, (cumulative) PT is by far the most influential model of framing. In essence, however, cognitive-formal accounts offer only a limited understanding of the source of framing effects. Usually, they fall short on offering an explanation for the specific form of the assumed functions, other than derivation based on data fitting (for an example of attempting to explain the source of probability weighting, see Brandstätter et al., 2002[9]).

### Emotional-motivational accounts

As soon as effects of attribute framing were found, it became increasingly clear that framing effects would be related to emotions. Asking “How dangerous is skiing?” presumably evokes different emotions than asking “How safe is skiing?” It is not evident how PT could be applied here. Therefore, it was argued that emotions might also be relevant for framing effects in risky choice framing. For instance, Schneider (1992[65]) proposed a motivational model, where two motives, security (the motive to avoid failure) and potential (the motive to succeed), jointly determine risk attitude. If, in a framing task, people were mainly motivated by security seeking, attention would be focused on avoiding the worst outcomes; if they were motivated by potential seeking, they would strive for attaining the best outcome. In a framing task, security seeking would lead to the choice of the sure gain, but also to the rejection of the sure loss since this option is unacceptable. Consequently, we would see a classic framing effect: preference for the sure gain, and the risky loss. This reasoning was later refined in regulatory-focus theory (Higgins, 2000[24]), arguing that self-regulation can be dominated by either the promotion focus (roughly: seeking positive outcomes), or the prevention focus (roughly: preventing negative outcomes). Kühberger and Wiener (2012[41]) applied this basic idea to a classic framing task as follows. Take three outcomes: a loss (simply quantified as (-1), a reference point outcome (0), and a gain (+1). Promotion-focused people focus on gains and tend to disregard other outcomes. This situation can be formalized as: -1 ≈ 0 < +1: the gain matters, other things don't. That is, gains are good, everything else is not good. Prevention-focused individuals, in contrast, focus on losses, thus: -1 < 0 ≈ +1. That is, losses are bad, everything else is less bad. The result? Under prevention focus (this motivation is assumed to be more frequent in people than is promotion focus; but note that focus can change situationally) people attend to the sure loss and avoid it - they therefore opt for the risky loss. No such tendency exists for gains, but a framing effect results when the tendency is strong enough for losses.

The above accounts arguably are more motivational than emotional. How could emotions come into play in risky choice framing tasks? According to Loewenstein et al. (2001[46]), emotions can influence decisions either as expected emotions when imagining the outcome of the decision, or immediately, contemplating the decision. Obviously, positive-negative framing can influence immediate emotions, that are existing when considering the current situation. The assumption here is that emotions mainly arise when considering the riskless part of the choice situation, with sure gains being emotionally attractive, and sure losses being emotionally aversive. Some research has collected direct evidence on this (e.g., Gosling and Moutier, 2017[21]; 2019[22]; Nabi et al., 2020[51]). Indirect evidence comes from a study by Kühberger and Gradl (2013[37]) who investigated the risky choice framing effect using a design where the options were evaluated in isolation, rather than in a choice context. They found that framing influenced the evaluation of only the riskless option but had little effect on the risky option. Given that framing mainly works on one constituent of the choice task (namely only the sure option), the distinction between risky choice, attribute, and goal framing tasks blurs. Rather, risky choice framing might be considered a special case of attribute framing. Some of the communicative accounts discussed below also imply that the framing effect is better construed as following from evaluative processes rather than from choice. 

### Communicative-pragmatic accounts

Researchers have increasingly doubted that decision making can be adequately understood by assuming that people are passive information processors, feeding in information as it is presented to them. Rather, people actively select and interpret information, and are often bringing background knowledge to bear. Often attention is focused on the described features of the task, and nothing else matters. Sometimes, however, information not explicitly presented, but considered relevant, is inferred. Our own framing research is an example of attentional focusing on what is explicitly presented (Kühberger, 1995[35]; Kühberger and Gradl, 2013[37]; Kühberger and Tanner, 2010[40]). Our argument is that in the classic risky choice framing task, the sure option (e.g., 200 people are saved) conveys only part of the truth, since it leaves open the complement about the remaining 400 lives. Note that only the sure option is described incompletely in classic framing tasks, while the risky option states both complements: e.g., that 600 people are saved with probability p, and that 0 people are saved with probability 1-p. The framing effect, then, could also follow from the lack of explication of the sure option. Indeed, adding the implicit complement to the sure option (e.g., 200 people saved *and 400 people not saved*), or deleting explicit complements of the risky option radically changes framing effects in ADP-like tasks. Specifically, completing the sure option, as well as subtracting parts of the risky option (e.g., withholding that *with 2/3 probability nobody will be saved*) makes the framing effect disappear. Playing around with adding and subtracting complements of the classic framing task has profound effects, reaching from increasing, to eliminating, and even reversing the classic framing effect (see also Mandel, 2001[48]). All this can be accomplished without changing the frame. Emotions are an obvious way to explain such effects: if the sure gain is only good, and the sure loss is only bad, the positive emotion with the good gain results in preference for the gain, and the negative emotion with the sure loss results in its rejection. If, however, the sure options are mixed (saved AND not saved; lost AND not lost), emotions do not longer provide a reason for preference.

Another ingenious account of framing is *Fuzzy-trace theory* (Reyna and Brainerd, 1991[58]). The basic argument is cognitive, but communicative-pragmatical aspects come into play in the framing task. Fuzzy-trace theory argues that people tend to process information on the most superficial and simple level. Thus, if possible, people simplify quantitative information to its qualitative gist. The original description *200 people will be saved* is simplified into *some people will be saved*. The same simplification is done with the risky option, presumably resulting in *some people will be saved or no one will be saved*. This simplified task contains no quantitative information, but it enables contrasting the simplified constituents: *some people will be saved* (sure gain) is contrasted to s*ome people will be saved* *or* *no one will be saved *(risky gain). In this contrast the winner is obvious: *some saved* is better than s*ome saved* *or* *no one saved. *Preference for the sure gain follows. In negative framing the same logic leads to the reversed preference: *some die* is worse than s*ome die* *or* *no one dies*. 

Still more radical accounts for framing effects deploy the pragmatics of language. In a most insightful paper Hilton (1995[25]) stressed that even in the experiment successful communication depends on some basic rules of communication. The most famous formulation of the rules enabling successful communication is Grice's (1975[23]) maxims, including the maxims of quantity, quality, relevance, and manner. In short, the maxim of quantity requires being as informative as necessary but not more; the quality maxim requires being truthful; relevance requires being on topic; and the maxim of manner requires being clear. Hilton's (1995[25]) argument is that in experiments researchers often fail to obey one or more of those maxims, thus leading experimental participants astray in their communication. Many effects in the so-called “heuristics and biases” program (Gilovich et al., 2002[19]) can be (at least partly) explained by violations of some of those maxims.

It is hard to see how risky choice framing effects can follow from direct misunderstandings in communication. However, they can follow indirectly, when pragmatical considerations come into play, by the sender or receiver of the framing message. The research of Sher and McKenzie (2006[68], 2011[69]) is instructive in demonstrating the role of the pragmatics of language for the framing effect. Their account proposes a specific form of information-leakage in framing tasks. Based on Hilton's (1995[25]) insight that human communication, even the logical vocabulary including conditionals, quantifiers, or probabilities, is not inherently disinterested, Sher and McKenzie argue for considering the reason why a communicator chooses to express an opinion in a specific frame. A speaker's choice of frame frequently is not random; rather it conveys implicit choice-relevant information. This can be about the quantity to be expected, or about the preference of the speaker him- or herself. Specifically, so the argument goes, speakers tend to choose descriptions in terms of some property when this property is above, rather than below, some expected reference point. For instance, describing a cup as being half full signals that it was expected to be empty (rather than full) in the given circumstances. In a similar vein, when it is communicated that 200 people are saved, it is implied that this is more than was expected. The receiver, by implication, understands that this option is good, because it is above expectation.

A related account was put forward by Moxey and Sanford (2000[50]). In their account, the focus is on the pragmatic interpretation of positive and negative natural language quantifiers (e.g., *many, a few, few*). Again, their use in communication entails some tacit implications, on what Moxey and Sanford call “reference set”, and “complement set”, respectively. They give the following example: In the expression *a few passengers were killed in the crash, which is a terrible thing*, the quantifier *a few* pertains to the passengers for whom the predicate is true, i.e., passengers who were killed. If it is said that *few passengers were killed in the crash, which is a good thing, *the quantifier pertains to passengers for whom the predicate is false, i.e., passengers who were not killed. In addition, it appears that positive quantifiers (e.g., all, some) tend to generate arguments in favor of the matter described, while negative quantifiers (e.g., none, not all) tend to generate arguments against the matter in question (Moxey and Sanford, 2000[50]).

Taken together, research has shown that there is more to framing tasks than meets the eye. Specifically, ordinary language operates on the assumption that expressions are chosen by good reason. If so, the choice of some expression is information that can be exploited to uncover reasons, or, in the case of framing, the likely attitude of the speaker (see also Teigen, 2015[72]; van Buiten and Keren, 2009[76]). In the end we arrive at a complicated and interrelated pattern for understanding framing effects. It is becoming increasingly clear that simple, one-dimensional explanations cannot do the job. Rather, framing effects can follow from: (i) a focus on different complements of an option; (ii) deep or shallow elaboration of the options; (iii) different psychophysical processes working on an edited input; and (iv) different motivational and emotional processes. Thus, there is no unitary source of framing effects, not even of the risky choice framing effect.

### Neural substrates of the framing effect

Some readers may wonder why nothing has been said about the neuroscientific evidence for framing. Indeed, one may have the hope that neuroscience may shed light on the role of the different processes implicated in framing. However, the available evidence falls short of this hope. First, the focus of neuroscientific research was limited to what was called here the emotional-motivational account, by arguing that the risky choice framing effect results when the framing differentially modulates emotional processes. Specifically, it was reported that frame-consistent choices were accompanied by increased amygdala activity, while frame-inconsistent choices were accompanied by increased activity in the dorsal anterior cingulate cortex (De Martino et al., 2006[11]; Roiser et al., 2009[61]). This made sense, since the amygdala is often associated with fear and anxiety, while the dorsal anterior cingulate cortex is associated with effortful control. Thus, framing was construed as following from a rapid emotional brain response that sometimes is corrected by effortful processing. More recent research comes to a different view: it is the right middle/frontal gyrus that is sensitive to valence framing, while the amygdala has no direct role in framing (Wang et al., 2017[77]). However, a comprehensive fMRI study combining actual with meta-analytic data championed another explanation: frame-consistent choices were correlated with resting brain activation, while frame-inconsistent choices were correlated with the task-engaged brain (Li et al., 2017[45]). This speaks against the interpretation of gain/loss framing as a competition between emotion and control. In sum, my reading of the neuroscience literature on framing does not enable an evaluation of the different accounts that have been put forward for framing effects.

## Framing Everywhere

The alert reader will have realized that the discussion of the framing effect was concentrated on experimental evidence collected in hypothetical scenarios. The ADP is prototypical example of this “imagine that” approach. Also clear is that the expressed preference will be of no consequence, since none of the options will be acted out. Framing research as reported here appears to be purely hypothetical. Is there also a real phenomenon? To investigate this question, researchers usually use gambles offering real gains and losses and frame them accordingly. Note, however, that it is difficult to recruit participants for a study where they face a chance to lose their own money. Therefore, the accepted paradigm is for the loss condition to offer an initial amount of money for real, say $6, and having participants lose money from this endowment. A gain condition then would be: Choose between A) winning $2 for sure; or B) winning $6 with probability ⅓ and $0 with probability ⅔. The loss condition would read: You get an initial endowment of $6. Choose between C) losing $4 with probability ⅔ and $0 with probability ⅓. This formulation ensures equivalence in terms of final outcomes. With outcomes being real gains and losses rather than framed gains and losses, if people show different preferences with gains and losses, this is called a *reflection effect*, rather than a framing effect. It appears that reflection effects are also reported frequently (for an overview see Oliver, 2018[54]).

By providing endowments, it thus is possible to do framing studies with real payoffs. Kühberger et al. (2002[38]) manipulated payoff type (real vs hypothetical) and payoff size (small vs medium) in a risky choice framing task and found the classic framing effect (risk-aversion for gains and risk-seeking for losses), but only for larger payoffs. With small payoffs, people chose the risk: the small payoff did not matter anyhow. Similar findings are reported for the reflection effect, even with larger real payoffs (e.g., Pommerehne et al., 1982[56]). Nevertheless, it is the essence of risky choice framing that it is “as if”, since, obviously, there are no repeated situations like the ADP. The framing only exists in the description, provided by language, while the actual outcomes are either gains, or losses. In the ADP, for instance, the outcomes are losses of human lives. Indeed, no single live can be gained, one can only avoid losing more lives.

### Framing animals

The animal literature on framing offers some inspiration on how it is possible to manipulate frames without relying on language. For instance, Marsh and Kacelnik (2002[49]), working with starlings, habituated the animals to expect either 1 (gains condition), or 7 pellets (loss condition) of food after pecking on a key. In the framing trials, the starlings could choose between either (a) always obtaining 4 pellets, or (b) obtaining 2 or 6 pellets of food with equal probability. The options (a) and (b) are outcome-equivalent, but differ in risk, since (a) always delivers the same outcome, while (b) offers two different outcomes, i.e., it is risky. Note that, if 1 pellet is expected after habituation, the choice between (a) and (b) is among two gain options. If, however, 7 pellets are expected after habituation, the choice between (a) and (b) is among two loss options. Marsh and Kacelnik (2002[49]) found a framing effect comparable to the experiments with humans with their starlings. Other animals also showed framing effects: bonobos and chimpanzees (Krupenye et al., 2015[31]); capuchin monkeys (Lakshminarayanan et al., 2011[42]); and rats (Bhatti et al., 2014[7]). Taken together, the framing effect seems to generalize to animal preferences, even though the framing manipulation is qualitatively different: animals are framed experientially by changing their expectation, while humans are framed by changing reference points by language.

### The broad concept of framing

The notion of framing is used very broadly. When I introduced the keyword “framing” in Google I got 305.000,000 hits by September, 2023. Even “framing and risk” delivered 74.000,000 hits. Obviously, then, the term has a wide range of applications. It is helpful to use the hierarchy proposed by Sher and McKenzie (2011[69]), to understand the broader meanings of the term. Sher and McKenzie (2011[69]) argue that framing manipulations can apply to different levels of equivalence. At the most basic Level 1, two utterances are truly equivalent. This applies if both (or all) frames supply identical evidence, and communication is disinterested. For instance, an attribute frame at Level 1 would require random selection of the description (e.g., 25 % success, or 75 % failure). Random selection of descriptions does rarely apply, thus Level 1 equivalence frequently is not attained. If descriptions are chosen deliberately, the frames are Level 2 information-equivalent: not disinterested, but logically equivalent. When frames are outcome-equivalent in the small world of economic analysis (what Mandel (2014[47]) calls the *proof-by-arithmetic argument*), but not in the enriched social world, Level 3 equivalence applies. This is the level of equivalence appropriate for the risky-choice framing task. At the lower levels 4 and 5, the framing notion gets a still more general touch. For instance, information summarized in lists, tables, or figures usually is Level 4 equivalent, since these descriptions summarize identical observations. For instance, in a table one can flip lines and columns, without changing the data. Or one can decide to report the results of a risky choice framing study in terms of percentage of people opting for the sure option, rather than the percentage opting for the risky option. By the way, there does not seem to be a consensus on what to report in framing studies, therefore care is needed in reading the results of framing studies. One could even argue that presenting the experimental instruction in native versus foreign language is a form of framing, equivalent at Level 4. Note also that research in risky decision making uses a variety of formats for presenting the risk information. An important distinction here is presenting the risk by description (the typical format in framing tasks) versus by experience (see Kühberger, 2021[34] for a discussion). Different probability formats could also be construed as a framing manipulation, Level 4. Finally, at Level 5 are emphasis-framing tasks. These are obviously not information-equivalent, since they highlight different aspects of topics. Thus “frames may be logically equivalent descriptions (Level 2), formally equivalent gambles (Level 3), observationally equivalent data digests (Level 4), or substantively equivalent attempts at persuasion (Level 5). But frames equivalent at Levels 2-5 are sometimes information non-equivalent at Level 1” (Sher and McKenzie, 2011[69], p. 53).

## Summary and Conclusion

This paper on framing started with the principle of description invariance. This principle requires that rational agents must show a consistent attitude to objects, regardless of their description, when those descriptions are co-extensive. Sounds quite reasonable, isn't it? It then reported on a variety of studies showing violations of description invariance in task descriptions that appear to be equivalent, at least superficially. These studies were characterized as either risky-choice framing, attribute framing, or goal-framing tasks, and an estimate of the size of framing effects found for the specific task types was provided. The framing effect size estimation for risky choice framing effects, i.e., the tendency to prefer the sure option with gains, and to select the risky option with losses, is d = 0.60, and this estimate is rather precise. It was also argued that the evidence for attribute framing is weaker, though conclusive. In contrast, there appears to be no goal framing effect, although much research has attempted to investigate this. I then described a variety of models accounting for the framing effect, grouped into cognitive-formal, emotional-motivational, and communicative-pragmatical models. Their merits were evaluated; the general take-home message being that the single reliance on PT to explain the framing effect is misguided. Why? Because the principle of description invariance is difficult to specify precisely. Indeed, it appears that the proof-by-arithmetic argument is inappropriate, and there are a wealth of further factors influencing preference in framing studies. Consequently, the verdict that a failure of invariance indicates irrationality stands on shaky ground. Rather, framing effects testify to the complexity of communication. Framing research does not show human irrationality, but something very different: that there are good reasons for the existence of framing effects if communication ought to be successful.

## Conflict of interest

The author declares no conflict of interest.
